# Foot Reflexology: Recent Research Trends and Prospects

**DOI:** 10.3390/healthcare11010009

**Published:** 2022-12-20

**Authors:** Deng-Chuan Cai, Ching-Yun Chen, Ting-Yun Lo

**Affiliations:** Graduate School of Design, National Yunlin University of Science and Technology, Yunlin 640301, Taiwan

**Keywords:** foot reflexology, bibliometric analysis, preventive medicine, corporate online open course, VOSviewer

## Abstract

Foot reflexology is a non-invasive complementary therapy that is increasingly being accepted by modern people in recent years. To understand the research trends and prospects of foot reflexology in the past 31 years, this study used the Web of Science core collection as the data source and two visualization tools, COOC and VOSviewer, to analyze the literature related to the field of foot reflexology from 1991 to 2021. This study found that the number of articles published in the field of foot reflexology has been increasing year by year, and the top three journals with the most articles are Complementary Therapies in Clinical Practice, Therapies in Medicine, and the Journal of Alternative and Complementary Medicine. The top three most prolific authors are Wyatt, Sikorskii, and Victorson, and the core institutions in the field of foot reflexology are Michigan State University, Northwestern University, Tehran University of Medical Sciences, and the University of Exeter. Foot reflexology has been shown to have a moderating effect on anxiety, fatigue, and cancer, and is a topic of ongoing and future research. This study uses this bibliometric analysis of foot reflexology literature to provide an overview of prior knowledge and a reference direction for modern preventive medicine.

## 1. Introduction

**Figure 1:**
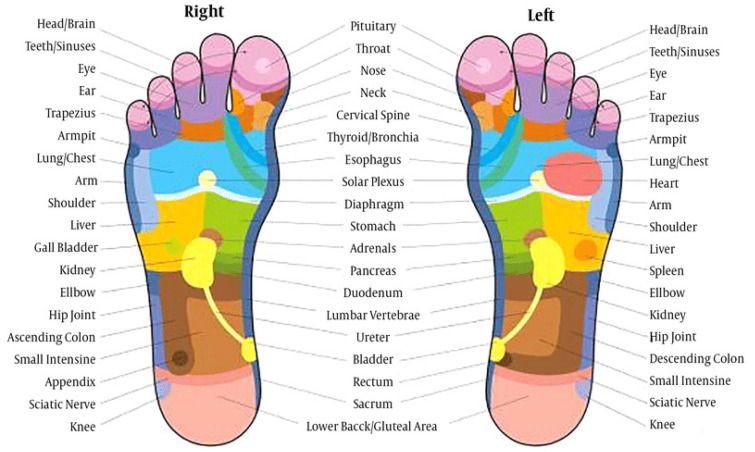
Foot reflexology chart.

Reflexology is an ancient therapy that was used by the early Chinese, Egyptians, and North American indigenous tribes for healing, but it was not until the 19th century that the medical community, nursing, and complementary and alternative medicine (CAM) practitioners began to explore it scientifically. In reflexology, there are five zones on each side of the body, each of which spreads down the arm and is confined to a single finger, while also extending down the body and leg until it aligns with a corresponding toe on the foot [1]. Reflexology is also known as regional therapy and reflex massage therapy. Its principle is that each area of the hands and feet represents each part of the body, such as the heart, liver, spleen, lungs, kidneys, and other internal organs, and when various areas of the hands and feet are massaged, it can stimulate energy, blood, nutrition, or nerves, resulting in therapeutic effects, including relieving mental stress, detoxifying the body, promoting blood circulation, losing weight, delaying aging, and improving internal health [2], as shown in Figure 1.

On the physical, emotional, and spiritual levels, reflexology often improves individuals’ physical health and well-being. Briefly, people choose reflexology because:

1. It does not use any drugs or chemicals and is the best choice for many health problems;
2. It can reduce pain;
3. It helps maintain physical flexibility and athleticism;
4. It relaxes the limbs, especially the hands and feet, and the whole body;
5. It stimulates the body to release pain-relieving chemicals;
6. It can be used as a means of preventing disease;
7. It promotes recovery from physical injury, especially for the hands and feet [3,4];
8. It triggers the release of endorphins and enkephalins, which help relieve pain and improve mental energy and well-being [5].

CAM can be divided into four types of treatment: (1) traditional Asian medical systems (TAMS), including traditional Chinese medicine, acupuncture, and acupressure; (2) alternative medicinal systems (AMS), including homeopathy and herbal therapy; (3) manual body-based therapies (MBBTs), including massage therapy, chiropractic therapy, osteopathy, and reflexology; and (4) mind-body therapies (MBTs), including hypnotherapy and psychotherapy [6,7]. Reflexology is one of the most commonly used modalities of CAM in Europe [8]. The most common reason for using CAM is dissatisfaction with the outcome of traditional medical care (CAM is most commonly used for musculoskeletal problems). A survey of reflexology use in the United Kingdom found an average one-year prevalence of 41.1% and an average lifetime use of 51.8% [9]. Usage rates for older adults in the United States range from 23% to 62.9% [10]. In Taiwan, the one-year usage rate is 85.65% [11]. In many countries, CAM is associated with the cosmetic industry and unorthodox medicine, and CAM is actively used in hospices, nursing homes, and obstetrics in healthcare settings. A study of clinicians showed that, on average, physicians consider CAM to be moderately effective, and 23.3% of them consider it to be an effective method of treatment [12] and would recommend it to their patients. The most common reasons for recommendation are to relax musculoskeletal pain, chronic disease, and nerve pain [13,14,15]. There is a worldwide shift in the attitude towards CAM due to its use as secondary care and integration into mainstream medical care [16]. In general, complementary and alternative medicine is increasingly recognized as a safe and effective way to reduce pain and illness [16]. It is also a non-invasive and inexpensive form of health care that can be used by most people (including children, the elderly, cancer patients, and pregnant women) [17]. Foot reflexology is one of the methods used by complementary and alternative medicine in medical treatment [1,18], and it differs from general foot massage as it targets the reflex zones of the feet corresponding to the body part in a deeper manner. Foot reflexology has been trained and certified by professional foot reflexologists as researchers [19,20], and it is commonly used in medical institutions as adjunctive therapy in an attempt to relieve pain or improve sleep when nurses are caring for patients.

Two internationally recognized reflexology methods are the Ingham method and the Rwo Shur method. The Ingham method is performed without using any tools, while the Rwo Shur method uses tools such as wooden sticks [4]. It is considered to be the most commonly used CAM for older people [21]. However, studies have also shown that younger people are more likely to use CAM than older people [22]. The founder of the Rwo Shur method (also known as Father Josef’s method), Father Josef Eugster Johann, suffered from osteoarthritis. After reading Gesund in die Zukunft (The Future of Health) by Schwester Hedi Masafret, he invented a new therapeutic method of foot reflexology to treat his osteoarthritis and suggested that “this home remedy could save medical expenses.” He founded the Fr. Josef Eugster Holistic Human Development (H.D.) Association and trained many foot reflexologists who have since helped scores of patients with stroke, insomnia, stomach pain, and neurological and skeletal disorders. Furthermore, the H.D. Association continues to explore reflexology clues in various parts of the body and develop new techniques that are useful in restoring health [23]. The World Health Organization (WHO) stated in 1990 that the combination of traditional drug-free podiatric medicine with modern surgical medicine can improve individuals’ understanding of their health while increasing medical coverage [24]. Thus, it is clear that foot reflexology is gradually being used as a non-invasive complementary therapy in medicine, nursing, and CAM practitioners to promote health restoration and the revitalization of various body parts at all ages as it has been proposed to enhance blood flow, relax the body, and improve injury healing [25].

Most clinical studies have found foot reflexology to be effective in reducing pain, including headache pain, back pain, joint pain [26], cesarean pain [27], acute pain in infants [28,29], pregnancy pain [30], labor pain [31,32,33,34,35,36], leg edema in pregnancy [37], postpartum women’s fatigue [38], pain from surgical medical examinations [39,40], organ removal or transplant pain [41,42,43,44], and various cancer pains [45,46,47,48,49]. It can also be used as a treatment for individual musculoskeletal cases [50]. In addition, foot reflexology is also an effective treatment for strokes, insomnia, asthma, diabetes, premenstrual syndrome, dementia, cancers, multiple sclerosis, and idiopathic detrusor overactivity [51,52,53]. It has also been studied for use in hospice units to alleviate discomfort and pain in patients at the end of life [54]. In fact, there are many studies exploring reflexology on the quality of life with cancer.

This study used bibliometric methods to uncover the development patterns in the field of foot reflexology research and to provide new academic perspectives and insights. To the best of our knowledge, no peer review published literature has summarized and analyzed the development of foot reflexology thus far. This study provides a comprehensive review of the research progress in the field of foot reflexology from its infancy to its peak between 1991 and 2021 (there has been research conducted prior to 1991 but it had poor methodology and publications) and offers insights into the prospects and opportunities for research in the field of foot reflexology. At the same time, the research results could facilitate the design and development of foot reflexology assistive tools and bring about a healthier and safer life for humans, giving greater value and energy to life.

## 2. Materials and Methods

### 2.1. Data Sources

In this study, the Web of Science (WOS) core collection was used to search the relevant literature in the field of foot reflexology. The search period covered a total of 31 years, from 1991 to 2021, and the process included an advanced search using TS = (“Foot massage” or “foot massage” or “reflexology”) as keywords to search all academic publications on topics related to foot reflexology. A total of 806 papers were retrieved, and 801 valid papers were obtained by importing the above keywords into COOC [55].

### 2.2. Analytical Methods

This study used the bibliometric software COOC version 12.6, which includes the abilities of data extraction, data cleaning, descriptive statistics, relationship construction, cluster mapping, theme evolution, and research frontiers, to analyze the data, and then imported the data into the VOSviewer visualization software (developed by Nees Jan van Eck and Ludo Waltman, researchers at Leiden University’s Center for Science and Technology Research; Leiden; Netherlands) for subsequent analysis [50]. The data were analyzed from the perspectives of the annual number of publications, journals, prolific authors, research institutions, authors and collaboration networks of research institutions, keywords, highly cited papers, and research fields, in order to provide an important reference for subsequent research and practitioners of foot reflexology [56].

## 3. Results

From 1991 to 2021, the Web of Science Core Collection collected 806 papers related to foot reflexology, of which there were 5 duplicates. The types of literature included 612 research papers, 107 review papers, and 82 papers of other types [55]. There were 414 publications in the field of foot reflexology, of which 94% were in English and 6% were in another language, including 1063 institutions in 62 countries, covering 75 research directions and 102 research areas [57].

The following subsections address the bibliometric profile of the literature on foot reflexology in terms of the annual number of publications, journals, prolific authors, research institutions, authors and collaboration networks of research institutions, keywords, and highly cited papers.

### 3.1. Annual Number of Publications

**Figure 2:**
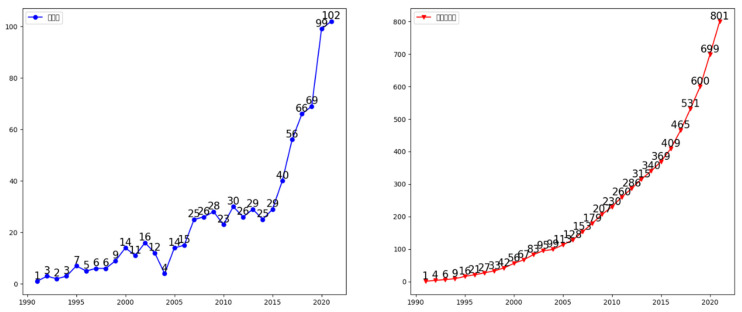
Distribution and cumulative volume of foot reflexology papers published over the years.

The evolution of the steady growth of foot reflexology-related research publications between 1991 and 2021 revealed that foot reflexology research results are increasing due to the advantages of foot reflexology as a complementary medicine and the findings and applications from relevant research (as shown in Figure 2) as well as journals accepting CAM reflexology articles for publication and an increasing number of researchers interested in CAM and reflexology.

The development of foot reflexology can be roughly divided into three stages, namely, the initial nascent stage, the primary growth stage, and the rapid development stage. The initial nascent stage (1991–2006) lasted for 16 years, with a total of 128 publications and an average of 8 publications per year. During the primary growth stage (2007–2015), scientific researchers began to show great interest in foot reflexology research, and the total number of publications grew to 241 publications, with an average of 27 publications per year. During the rapid development stage (2016–2021), the number of annual publications accelerated, with a total of 434 publications accumulated over six years and an average of 72 publications per year, representing nine times the amount of research performed during the primary growth stage. This study of CAM and foot reflexology found that the significant papers in the field were mainly concentrated in the initial nascent stage (eight papers in total, accounting for 53% of the number of significant papers), and the remaining seven papers (47%) were concentrated in the primary growth stage. The results of previous studies, therefore, laid the foundation for future studies. The rapid turnover of research papers published in the third stage, with the number of papers exceeding 102 for the first time in 2021, showed a trend of diversified growth in the field of foot reflexology and its richer research content, indicating the extensive and prominent importance of this field in the world scientific community and reflexology practitioners.

### 3.2. Journals

In this study, by counting the number of foot reflexology papers in the Web of Science Core Collection against foot reflexology source publications, it was found that 801 papers were published in 414 journals during the past 31 years. The top 10 countries in terms of the number of papers issued were the United States (33.3%), the United Kingdom (23.9%), the Netherlands (4.1%), Switzerland (3.9%), Germany (3.6%), Iran (3.6%), Turkey (3.6%), Canada (3.1%), Australia (2.9%), and India (2.9%). Taiwan (0.5%) ranked 14th among the 28 countries in which the papers were published. Among the top 15 journals with the most published articles, Complementary Therapies in Clinical Practice accounted for 11.1%, Complementary Therapies in Medicine accounted for 9.6%, the Journal of Alternative and Complementary Medicine accounted for 6.30%, Evidence-based Complementary and Alternative Medicine accounted for 3.90%, and the Cochrane Database of Systematic Reviews accounted for 3.10%, which together accounted for one-third of the total number of published articles. The Journal Citation Indicator (JCI) values for these journals fell between 0.73 and 1.31, indicating the average citation impact of the articles published over the past three years was above the standard. In 2020, the journal ranking where the foot reflexology papers were published was between Q1-Q2, and the influence factor is in the top 25–50%. The top three publishers were Elsevier (27.4%), Wiley (11.7%), and Lippincott Williams & Wilkins (6%). The top 15 journals publishing the most articles on foot reflexology are listed in Table 1.

**Figure 3:**
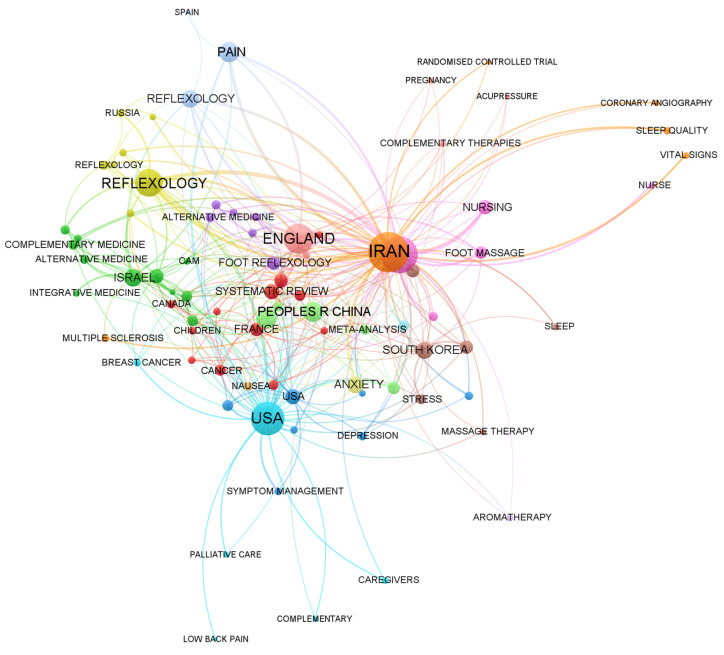
Clusters of similar topics in foot reflexology studies in countries around the world (conditions are calculated with a keyword frequency ≥ 5 and a country frequency ≥ 10).

**Figure 4:**
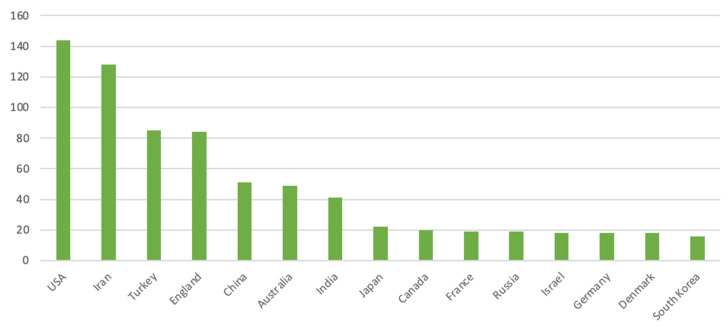
The top 15 countries for foot reflexology paper production.

To identify the main countries and regions involved in the field of foot reflexology as well as international collaborations, this study visualized the interactions between countries and regions. Figure 3 shows the geographical distribution of the main countries worldwide and the knowledge domains of the co-authorization. This research found 62 countries around the world participating in the study of reflexology. The nodes represent different countries, while the sizes of the nodes represent the domain activity and scientific output of the country. The various colors represent different clusters of the co-location matrix based on the corresponding countries. The connection between the two nodes indicates their knowledge collaboration. The thickness of the lines indicates the degree of collaboration. Obviously, the stronger the connection between two nodes, the deeper the contact and cooperation between the two countries. From the figure, 12 clusters could be seen. Figure 4 shows the top 15 countries with the most published papers, among which the top three countries were the United States, Iran, and Turkey, and Table 2 presents the related research in each country, which mostly focuses on the application analysis of foot reflexology. As can be seen from the following graphs, a great deal of effort and cooperation in the progress of foot reflexology research has been conducted in every country in the world. This indicates that the future trend in this field is for many countries and regions to value and expect more diversified cooperation and development.

### 3.3. Prolific Authors

**Figure 5:**
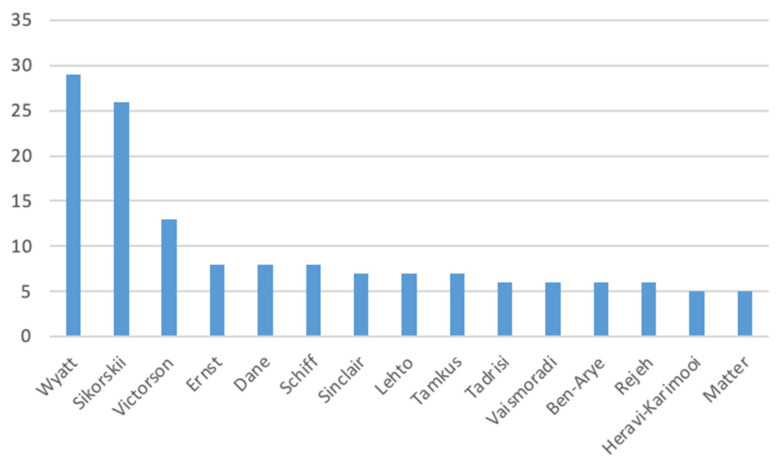
Author rankings related to foot reflexology.

The foot reflexology research during this time frame covered 140 categories, and there were more than 2716 authors in the Web of Science Core Collection. The top 15 authors with the most publications are shown in Figure 5, with the top three authors being Wyatt, Sikorskii, and Victorson. Wyatt, Sikorskii, and Victorson have published 13 papers together, and Wyatt and Sikorskii have published 26 papers together. Wyatt began his research on reflexology for cancer and family caregivers in 2006 [46,49]. He collaborated with Sikorskii and Victorson on reflexology for cancer symptom management in 2007 and explored methodologies in CAM intervention trials in 2009–2010. He began his research on reflexology for advanced breast cancer in 2012–2013, studied clinical trials on caregiver-provided reflexology in 2015–2017, and began a number of ongoing randomized controlled trials for symptom management through family caregiver-provided reflexology for patients with advanced breast cancer in 2018–2021. Today, Wyatt is committed to researching the pain and psychological impact of reflexology on women with advanced breast cancer. As such, foot reflexology has progressed to the level of research that enhances patients’ psychological comfort to reduce pain.

### 3.4. Research Institutions

This study analyzed important research institutions studying foot reflexology, which could help researchers to gauge the important global research power and make an integrated consideration. After the analysis, 1183 research institutions involved in the field of foot reflexology were found, and the top 15 research institutions are shown in Table 3. In this study, the institutional attributes of the top 15 research institutions were obtained by a Google query. From 1991 to 2021, Michigan State University (the United States) appeared the most frequently (32 times), which was twice as often as Northwestern University (USA) in second place (15 times). The top 15 institutions were mostly medical universities (53%), research universities (40%), and government units (7%). The top three institutions in terms of independent journal publication capacity were Michigan State University (11 articles), which focused on breast cancer patients and reflexology, followed by the All India Institute of Medical Sciences (8 articles), which focused on epilepsy patients. The third place was taken by Iran’s Tabriz University of Medical Science (6 articles), which studied multiple applications of reflexology (including elderly sleep, women in labor, and adult coronary artery surgery). This study found that inter-institutional collaborations and co-publications were common, with a maximum of 16 institutions and a minimum of 2. The mode of collaboration was mostly a consortium of national university research institutions, but multinational research institutions and banks were also involved.

### 3.5. Collaborative Network of Prolific Authors and Research Institutions

**Figure 6:**
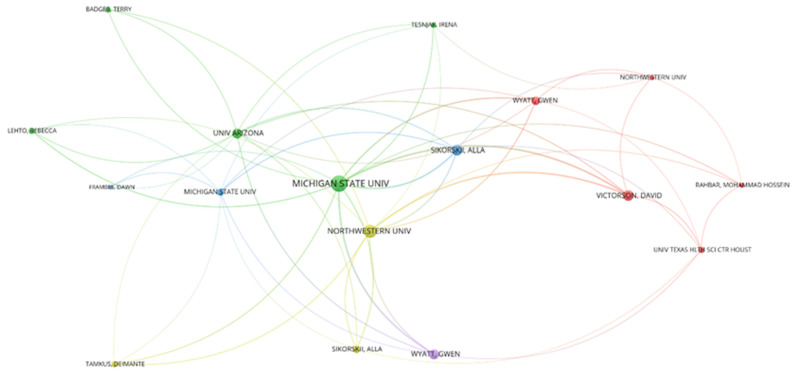
The collaborative network of prolific authors and research institutions in the field of foot reflexology research between 1991 and 2021.

There were a number of closely collaborating author clusters within prolific authors and institutions with strong subcluster ties, as illustrated by the red, blue, and yellow clusters, using papers by Victorson (a medical scientist in the United States), Sikorskii (a psychiatry researcher in the United States), and Wyatt (a nursing researcher in the United States) as the core of their papers. The thickness of the line in Figure 6 represents the frequency of collaboration. The same color of a line means the same research topic, and the color change of a line between two points means that after collaborating for a period of time, the research direction changed. If the colors are similar at first, it means the research topic is highly similar. The red dots represent the three authors, Victorson, Wyatt, and Rahbar, who collaborated most closely with Northwestern University and the University of Texas Health Science Center at Houston on the topic. The blue dots represent collaborations between Sikorskii, Frames, and Michigan State University. The green dots represent Badger, Lehto, and Tesnjak’s collaborations with Michigan State University and the University of Arizona. The yellow dots represent Sikorskii and Tamkus’ affiliation with Northwestern University. The purple dots represent Wyatt’s cross-collaboration with Michigan State University across different research themes. The same authors worked on different areas of foot reflexology [57]. The collaborative network of highly productive authors and research institutions is shown in Figure 4.

### 3.6. Popular Keywords

**Figure 7:**
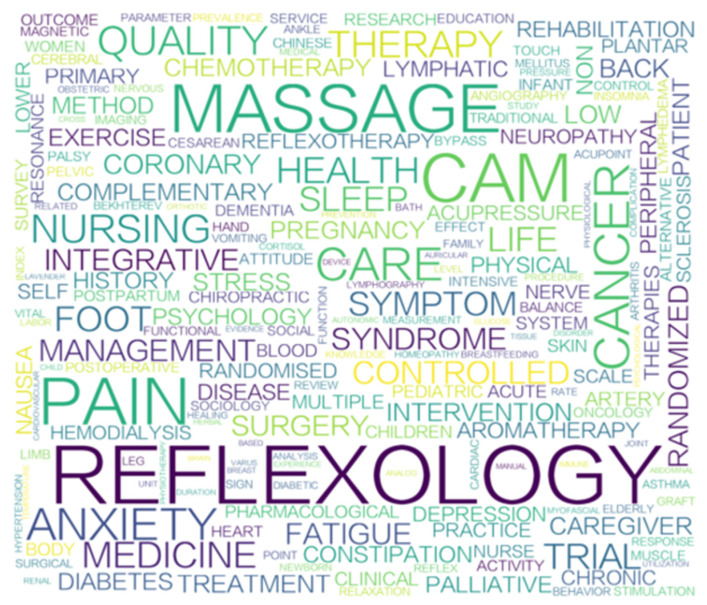
Reflexology keyword cloud.

Out of the word cloud created from the keywords used during the last 31 years, the top 15 high-frequency words were reflexology, CAM, massage, pain, anxiety, nursing, quality of life, cancer, fatigue, breast cancer complementary medicine, pregnancy, multiple sclerosis, constipation, and chemotherapy [59], as shown in Figure 7.

#### 3.6.1. Evolution of Keywords over the Years

In this study, the evolution of the keywords related to reflexology papers was studied by filtering 10 keywords for each year under the condition of an occurrence frequency ≥ 5. From the results of the analysis, the top three keywords accumulated over the 31 years were reflexology, massage, and complementary medicine. The term “alternative medicine” appeared for the first time in 1995 [60]; “massage” appeared for the first time in 1996; “foot reflexology” appeared for the first time in 1997; and “complementary medicine” appeared for the first time in 1998. Next, the most common keyword-related topics were anxiety, with 45 papers mentioning this keyword for 11 consecutive years out of the 31; quality of life, with 23 papers listing it for 8 out of 31 years; fatigue, with 19 papers listing it for 11 out of 31 years, with 19 papers; and sleep, with 14 papers listing it for 7 out of 31 years. Among the disease categories, reflexology was mainly investigated in relation to cancer, chemotherapy, lower back pain, hemodialysis, constipation, coronary angiography, and other topics. In the discussion related to women, foot reflexology for pregnancy was used as a keyword continuously for 9 out of 31 years, with 12 papers, while foot reflexology for breast cancer was used as a keyword continuously for 7 years out of 31 years, with 8 papers total. The development of foot reflexology is described below. In the initial nascent stage (1991–2006), alternative therapies, massage, and foot reflexology generated the first concepts and theoretical discussions. It was not until the primary growth phase (2007–2015) that quality of life, anxiety, fatigue, sleep, pregnancy, and breast cancer began to be studied as topics. In recent years, during the rapid growth phase (after 2016), research related to foot reflexology has expanded to explore its role in CAM and related applications, including hemodialysis, prosopagnosia, constipation, chest pain, intensive care unit case symptom management, and comparison of pre- and post-surgical intervention trials. In addition, this study found annual publications in the field of foot reflexology containing topics such as massage, anxiety, fatigue, and cancer over a five-year period from 2017–2021.

#### 3.6.2. Keyword Co-Occurrence Clustering

**Figure 8:**
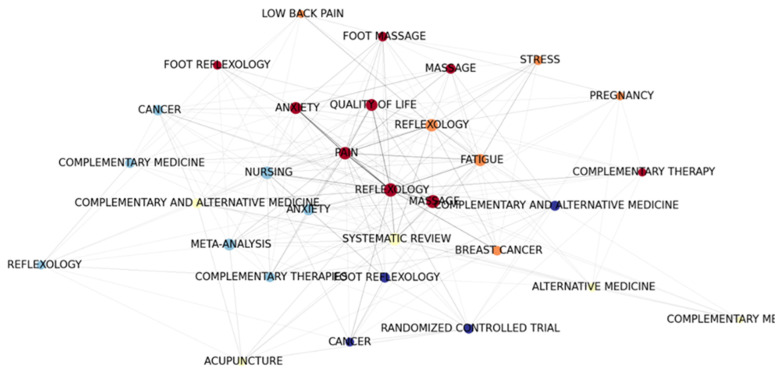
Reflexology keyword co-occurrence clustering graph.

From the reflexology keyword co-occurrence clustering, five clusters of related studies could be found. In the reflexology keyword co-occurrence clustering map shown in Figure 8, these clusters are presented in different colors: (1) orange, representing foot reflexology and female status; (2) red, representing foot reflexology and quality of life; (3) light blue, representing foot reflexology as CAM; (4) dark blue, representing foot reflexology and cancer research; and (5) light yellow, representing foot reflexology and CAM system evaluation.

### 3.7. Highly Cited Papers

Highly cited papers tend to be published in high-impact journals (journals with high average citation rates), reflecting the influence and trends of the field [61]. This study found 15 highly cited papers in foot reflexology research (with ≥ 100 citations), as shown in Table 4. Most of the highly cited papers with more than 100 citations were published during the initial nascent stage (1999–2005, eight papers) and the primary growth stage (2006–2015, seven papers), which laid the cornerstone for the later rapid development of foot reflexology.

### 3.8. Future Research Directions

**Figure 9:**
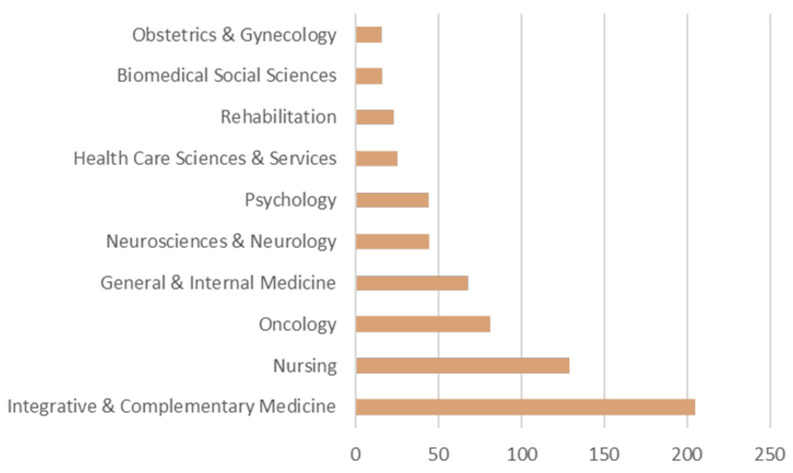
Top 10 research areas for foot reflexology.

According to Clarivate Analytics’ Web of Science classification, most publications in the field of foot reflexology are related to ten major categories: integrative complementary medicine; nursing; oncology; medicine, general and internal; neurosciences and neurology; psychology; health care sciences and services; rehabilitation; biomedical social sciences; and obstetrics and gynecology, as shown in Figure 9. In terms of the number of publications, these ten major categories are the main directions of future foot reflexology research. The ten research directions account for 61% of the publications related to foot reflexology, indicating that the applications of foot reflexology are no longer a single direction of research but focus more on the breadth and depth of research in medical, nursing, and psychological applications. At the same time, these directions are also the future research trends of preventive medicine [7,62].

## 4. Discussion

This study used COOC and VOSviewer bibliometric software to analyze the annual number of publications, journals, prolific authors, research institutions, authors, and collaboration networks of research institutions, keywords, highly cited papers, and research fields. It explored the research progress and the lineage of foot reflexology during the 31 years from 1991 to 2021 [57,63]. This study was based on a literature review and comparison with research results that could be used as a basis for the future development of the field of foot reflexology [56]. The results of the analysis are summarized below.

1. The research publications on foot reflexology from 1991 to 2021 could be divided into three stages: (1) the initial nascent stage; (2) the primary growth stage; and (3) the rapid development stage. As for the current phase of rapid development, the number of publications has increased year by year, from 1 paper in 1991 to 102 papers in 2021, an increase of 102 times, indicating that academics have begun paying more attention to foot reflexology.
2. Complementary Therapies in Clinical Practice is the most published journal, and although its influence factor was not the first among the top 10 journals, it has been gradually gaining attention and influence over the past 31 years. The influence factor of the Cochrane Database of Systematic Reviews was 9.289, indicating the high quality of its included papers and its strong influence in the field of foot reflexology, as well as its importance as a source of information for research on foot reflexology. Another factor to consider is that researchers are now wanting to research reflexology and journals are more willing to publish CAM research.
3. Wyatt, Victorson, and Sikorskii are among the more active scholars in the field of foot reflexology research publications and they have established numerous extensive collaborations.
4. Michigan State University, Northwestern University, the University of Exeter, and the University of Tehran were found to be the core institutions for foot reflexology research.
5. Regarding the prolific authors and collaboration networks of research institutions, this study found that Victorson, Wyatt, and Rahbar have worked closely with Northwestern University and the University of Texas Health Science Center at Houston; Sikorskii and Frames have worked closely with Michigan State University; and Badger, Lehto, and Tesnjak have worked closely with Michigan State University, which is the largest institution in terms of cross-collaboration across different research themes. Michigan State University is the largest institution in terms of cross-collaboration across different research themes, with strong cross-collaboration often occurring between different authors and institutions.
6. According to the statistics of the authors’ keyword usage, the top 15 keywords were reflexology, CAM, massage, pain, anxiety, nursing, quality of life, cancer, fatigue, breast cancer, complementary medicine, pregnancy, multiple sclerosis, constipation, and chemotherapy.
7. The number-one cited paper was published in 2001 by Thomas, Nicholl, and Coleman and titled “Use and Expenditure on Complementary Medicine in England: A Population Based Survey”, which has been cited 451 times.
8. Research on foot reflexology involves research in integrative complementary medicine, nursing, oncology, medicine, neuroscience and pathology, psychology, health care science and services, rehabilitation, sociobiological sciences, and obstetrics and gynecology from a whole-person care perspective.

### Limitations and Recommendations

Although this study analyzed the current state of research on foot reflexology, limitations remain. The limitations and recommendations of this study are as follows.

1. The search was mainly focused on the Web of Science; Web of Science was used because it is a popular scholarly search system for experts and researchers, and it has numerous high-quality publishing houses and academic papers.
2. Web of Science did not find any of the studies on labor duration and pain which is a limitation.
3. In this study, only the annual number of publications, journals, prolific authors, research institutions, authors and collaboration networks of research institutions, keywords, highly cited papers, and research fields were considered. Future studies should develop more diversified directions or do a cross-analysis of empirical research cases. At the same time, interviews with experts, technicians, and people who have received foot reflexology should be conducted to compare the differences between foot reflexology and traditional foot massage, as the design and development of foot reflexology hand tools could be promoted by understanding the feelings of the recipients. All of the above will lead to more breakthroughs and discoveries in foot reflexology, which is expected to bring more multidimensional thoughts and provide cross-disciplinary support to human beings.

## 5. Conclusions

This study was a bibliometric analysis of 31 years of literature in the field of foot reflexology from 1991 to 2021. A substantial literature review and analysis were conducted to understand the past, present, and future directions of foot reflexology. This study found that the top three most productive journals are Complementary Therapies in Clinical Practice, Therapies in Medicine, and the Journal of Alternative and Complementary Medicine; the top three most prolific authors are Wyatt, Sikorskii, and Victorson; and the core institutions in the field of foot reflexology are Michigan State Univ, Northwestern Univ, and Univ Tehran Med Sci, Univ Exeter.

Foot reflexology practitioners are continuing to study the pathological characteristics of the foot in relation to various parts of the body while exploring the fluency and precision of the techniques being used. It is also expected that the concept of foot reflexology will be introduced to family members to protect the health of the whole family and to provide physical and psychological relief to patients with serious clinical conditions. Furthermore, this study aimed to bring more attention to the use of foot reflexology as a CAM modality in preventive medicine in the future. These healthcare methods can become a part of people’s lives and give them a more solid, comprehensive, and healthy sustainable life value.

## Figures and Tables

**Table 1:** The top 15 journals publishing the most articles on foot reflexology (in order of the total number of publications).

Journal Title	Issuing Country	Quantity Published	Percentage	2020 Influence Factor	2020 JCI [58]	Total Citations	2020 Ranking
Complementary Therapies in Clinical Practice	Netherlands	46	11.1%	2.446	0.82	2275	Q2
Complementary Therapies in Medicine	Netherlands	40	9.6%	2.446	0.82	2275	Q2
Journal of Alternative and Complementary Medicine	United States	26	6.3%	2.582	0.89	5858	Q2
Evidence-based Complementary and Alternative Medicine	United Kingdom	16	3.9%	2.63	0.73	22,229	Q2
Cochrane Database of Systematic Reviews	United Kingdom	13	3.1%	9.289	1.31	81,217	Q1
Oncology Nursing Forum	United States	12	2.9%	2.172	0.76	4230	Q2
European Journal of Integrative Medicine	Germany	12	2.9%	1.314	0.51	1301	Q4
Holistic Nursing Practice	United States	10	2.4%	1	0.56	923	Q3
Journal of Advanced Nursing	United Kingdom	9	2.2%	3.187	1.59	23,215	Q1
Psycho–Oncology	United Kingdom	9	2.2%	3.894	1.02	15,157	Q2
Supportive Care in Cancer	United States	8	1.9%	3.603	1.12	18,290	Q1
Journal of Korean Academy of Nursing	Korea	8	1.9%	0.984	0.38	1229	Q4
International Journal of Nursing Practice	Australia	8	1.9%	2.066	0.93	2676	Q2
Alternative Therapies in Health and Medicine	United States	8	1.9%	1.305	0.36	1372	Q4
European Journal of Oncology Nursing	United States	8	1.9%	2.398	0.98	3404	Q2

There are 414 journals in total, and the top 15 are listed in the table above.

**Table 2:** Clusters of published papers by country and research topic.

Cluster	Country	Topics
1	Australia, Canada, and France	Cancer, children, coronary artery disease, and rehabilitation
2	Denmark and Israel	Alternative, complementary, and homeopathic remedies
3	The United States	Pain, breast cancer, depression, hemodialysis, and symptom management
4	Japan and Russia	Foot reflexology, massage therapy, cerebral palsy, and constipation
5	India	Complementary therapies and anxiety
6	The United States	Caregivers, complementary therapies, lower back pain, breast cancer, and palliative care
7	Iran	Coronary angiography, multiple sclerosis, vital signs, sleep quality, and randomized controlled trial
8	South Korea	Fatigue, massage therapy, quality of life, sleep, and stress
9	Turkey	Chemotherapy, foot reflexology, and care
10	The United Kingdom	Pregnancy and complementary therapy
11	China	Massage and meta-data analysis
12	Spain	Pain and foot reflexology

**Table 3:** The top 15 research institutions in terms of the number of articles published on foot reflexology.

Ranking	Institute	Country	Percentage	Number of Independent Publications	Number of Articles Published	Cooperative Institutes	Remarks
1	Michigan State Univ	United States	2.70%	11	32	16	(public) research university
2	Northwestern Univ	United States	1.30%	1	15	11	(private) research university
3	Univ Exeter	United Kingdom	0.90%	4	11	7	(public) research university
4	Univ Tehran Med Sci	Iran	0.90%	3	11	15	(public) medical university
5	Univ Ulster	United States	0.85%	3	7	6	research university
6	Univ Arizona	United States	0.76%	0	9	6	(public) medical university
7	All India Inst Med Sci	India	0.76%	8	9	2	(public) medical university
8	Mashhad Univ Med Sci	Iran	0.76%	0	9	15	(public) medical university
9	Baqiyatallah Univ Med Sci	Iran	0.76%	2	7	9	(public) special medical university
10	Ataturk Univ	Turkey	0.68%	1	8	4	(public) research university
11	Univ Liverpool	United Kingdom	0.68%	1	8	11	research university
12	Tabriz Univ Med Sci	Iran	0.68%	6	8	4	(public) medical university
13	Univ Hull	United Kingdom	0.68%	1	8	7	medical university
14	Minist Hlth	Israel	0.68%	0	8	6	government health unit
15	Shiraz Univ Med Sci	Iran	0.68%	4	8	7	(public) medical university

**Table 4:** The top 15 highly cited papers in foot reflexology (cited ≥ 100 times).

Number	Year of Publication	Title	Journals	Citation Frequency
1	2001	Use and Expenditure on Complementary Medicine in England: A Population-based Survey	Complementary Therapies in Medicine	451
2	2000	The BBC Survey of Complementary Medicine Use in the UK	Complementary Therapies in Medicine	303
3	2007	Nasal Saline Irrigations for the Symptoms of Chronic Rhinosinusitis	Cochrane Database of Systematic Reviews	219
4	2005	Use of Complementary and Alternative Medicine in the Scandinavian Countries	Scandinavian Journal Of Primary Health Care	157
5	2001	Alternative Therapies Among Adults With a Reported Diagnosis of Asthma or Rhinosinusitis—Data from a Population-Based Survey	Chest	144
6	2011	Diagnosis and Treatment of Plantar Fasciitis	American Family Physician	125
7	2008	Plantar Fasciitis: Evaluation and Treatment	Journal of The American Academy of Orthopaedic Surgeons	121
8	2013	Prevalence of Use of Complementary and Alternative Medicine (CAM) by Patients/Consumers in The UK: Systematic Review of Surveys	Clinical Medicine	121
9	2013	Non-pharmacological Interventions for Fatigue in Rheumatoid Arthritis	Cochrane Database of Systematic Reviews	120
10	2000	Complementary Therapies: Have They Become Accepted in General Practice?	Medical Journal of Australia	115
11	2008	Quantitative Lymph Imaging for Assessment of Lymph Function Using Indocyanine Green Fluorescence Lymphography	European Journal of Vascular and Endovascular Surgery	114
12	2001	Nasal Irrigation for the Alleviation of Sinonasal Symptoms	Otolaryngology–Head And Neck Surgery	108
13	2004	Breast Cancer Patients Have Improved Immune and Neuroendocrine Functions Following Massage Therapy	Journal of Psychosomatic Research	105
14	2014	Summary of Evidence-based Guideline: Complementary and Alternative Medicine in Multiple Sclerosis: Report of The Guideline Development Subcommittee of The American Academy of Neurology	Neurology	103
15	2005	The Integration of Complementary Therapies in Australian General Practice: Results of a National Survey	Journal of Alternative and Complementary Medicine	100
